# Magnetic properties of N-doped graphene with high Curie temperature

**DOI:** 10.1038/srep21832

**Published:** 2016-02-24

**Authors:** Qinghua Miao, Lidong Wang, Zhaoyuan Liu, Bing Wei, Fubiao Xu, Weidong Fei

**Affiliations:** 1 School of Materials Science and Engineering, Harbin Institute of Technology, Harbin 150001, China

## Abstract

N-doped graphene with Curie temperature higher than room temperature is a good candidate for nanomagnetic applications. Here we report a kind of N-doped graphene that exhibits ferromagnetic property with high Curie temperature (>600 K). Four graphene samples were prepared through self-propagating high-temperature synthesis (SHS), and the doped nitrogen contents of in the samples were 0 at.%, 2.53 at.%, 9.21 at.% and 11.17 at.%. It has been found that the saturation magnetization and coercive field increase with the increasing of nitrogen contents in the samples. For the sample with the highest nitrogen content, the saturation magnetizations reach 0.282 emu/g at 10 K and 0.148 emu/g at 300 K; the coercive forces reach 544.2 Oe at 10 K and 168.8 Oe at 300 K. The drop of magnetic susceptibility at ~625 K for N-doped graphene is mainly caused by the decomposition of pyrrolic N and pydinic N. Our results suggest that SHS method is an effective and high-throughput method to produce N-doped graphene with high nitrogen concentration and that N-doped graphene produced by SHS method is promising to be a good candidate for nanomagnetic applications.

Graphene has attracted tremendous attention since its first isolation by Novoselov and Geim in 2004^1^,^2^. It has been shown that graphene has many excellent properties in extensive fields such as energy materials, microelectronics, sensors and superconductors to be expected^3^,^4^. In recent years, researchers found magnetism in doped or defective graphene or graphene oxide^5^,^6^,^7^, which has inspired the widespread interest in the origin of magnetism, influencing factors and prospective application of these 2D materials.

Magnetism in nanomaterials is a scientific discipline at the forefront of fast emerging fields in nanoscience and nanotechnology. In current technological applications, magnetic materials are mainly based on *d* and *f* elements. Unexpected magnetic properties have been found in some low dimensional materials. The reduction in one or more dimensions typically results in the reduction of the coordination number of atoms, which reduces the hopping tendency of electrons^8^,^9^,^10^. Furthermore, the Coulomb interaction/bandwidth ratio is expected to be enhanced, which facilitates the tendency towards the appearance of magnetism in materials with reduced dimensions.

Studies on the magnetic properties of graphene have established the possibilities of developing magnetic materials with lightweight, high strength and high thermal conductivity. It has been recently shown that carrier-doped graphene has a very large diamagnetic susceptibility^11^. The susceptibility decreases rapidly with increasing carrier-doping of either electrons or holes^12^. Chen and Oleg V. Yazyev have reported that the modification of graphene with point defects leads to the implementation of magnetism based on carbon nanostructures in which a ferromagnet-antiferromagnet transition is possible^13^,^14^. A small ferromagnetic signal at room temperature in hydrogen terminated graphene prepared by Birch reduction of graphite oxide has also been observed with a magnetization of 0.006 emu/g^15^. Similarly, a room temperature saturation magnetization of 0.02 emu/g was reported after partial reduction of graphene oxide using hydrazine^16^. Samples consisting of reduced graphene oxide have been reported to have a magnetization of 0.79 emu/g at 300 K and the value increased to 1.99 emu/g by further annealing at 500 °C^17^. It is interesting to find that high room temperature ferromagnetic moment with high Curie temperature (>700 K) for graphene oxide (GO) is obtained by a simple chemical activation using phosphoric acid followed by heat treatment, while its coercivity is less than 20 Oe^6^. Substitutional doping is a promising way to modulate the electronic and magnetic properties of graphene^12^. It has been reported that the room temperature magnetization of graphene embedded carbon (GSEC) films after 100 eV low-energy electron irradiation can be up to 0.26 emu/g^18^. It has also been reported that N-doped graphene can be synthesized through vacuum annealing a sandwiched substrate at high temperature^19^. Du *et al*. have prepared N-doped graphene by annealing reduced graphene oxide in ammonia, which can increase its magnetization at a relatively low temperature (≤600 °C)^20^. Li *et al*. pointed out that pyrrolic N could induce a net magnetic moment of 0.95 μ_B_/N, compared with pyridinic N which has less influence on the spin polarization of the edge states^21^. A synthetic route based on a stoichiometric dehalogenation of perhalogenated arene and pyridine precursors by a transition metal enables the formation of sp^2^-coordinate carbon with graphene domains and the option to incorporate nitrogen especially on pyrrolic bonding sites^22^. A Curie temperature of approximately 100 K and magnetization of 1.66 emu/g at 2 K were reported for N-doped GO^23^. The pyrrolic N-doped graphene synthesized through a high-throughput hydrothermal method with 6.02 at.% doping concentration exhibited significant ferromagnetism with a saturation magnetic moment (0.014 emu/g) and a narrow coercivity (181.4 Oe)^5^. Consequently, the magnetism of graphene is a hot research topic due to the interesting properties and several advantages over the conventional transition metal based ferromagnetism.

The magnetic properties in graphene and related 2D carbon materials are often explained by the existence of different kinds of defects^14^, structural disorder, dangling bonds or carbon edge termination^13^,^24^,^25^,^26^. It is generally accepted that ferromagnetism in graphene systems is caused by an indirect coupling between localized magnetic moments within the materials, mediated by the charge carriers^27^,^28^,^29^,^30^,^31^,^32^,^33^. This kind of coupling, known as Ruderman-Kittel-Kasuya-Yosida (RKKY) interaction, presents a unique behavior in graphene, unlike the discovery in metallic two-dimensional system^34^. Moreover, the oscillatory properties of RKKY coupling between on-site impurities in graphene are ruled by the principle relating the sign of the coupling integral to the sub-lattice in which both magnetic impurities are placed^35^,^36^,^37^. And these systems include nanoflakes with zigzag and armchair edges and equal number of carbon atoms in two sub-lattices as well as graphene with both kinds of edges^38^,^39^,^40^. The defects in the dense areas are expected to contribute to the ferromagnetic coupling while the number of defects located on the neighboring sites is supposed to increase with increasing the density of defects. Thus, defects could induce localized moments and play an important role in the ferromagnetism of graphene. However, the long range interactions between these localized moments which give rise to ferromagnetism are still controversial. It is not clear why there are such strong interactions in these N-doped samples, which give rise to high Curie temperatures. In addition, although the magnetic of graphene based materials have been studied extensively, ferromagnetic graphene materials with a Curie temperature much higher than room temperature and higher coercive forces are rare.

Self-propagating High-temperature Synthesis (SHS) has received considerable attention since it is a relatively simple, fast, low-cost, and efficient novel material production method^41^. It has also been used to produce certain advanced ceramic, composites, intermetallic compounds and carbon nano-tube^42^,^43^,^44^. As an alternative to conventional furnace technology, SHS is usually meant an exothermal reaction caused by a short thermal pulse (ignition) and then propagates due to intense heat release and heat transfer from hot to cold parts, forming a combustion wave. The temperature of the combustion can be very high (as 5000 K) and the rate of wave propagation can be very rapid (as 25 cm/s), hence this process offers the opportunity to investigate reactions in conditions of extreme thermal gradients (as 10^5 ^K/cm). SHS is widely applied in producing various materials, including carbon-free, this method has not yet been applied to synthesizing CNTs^45^; It has been known that magnesium can act as a carbon reducer to produce various solid-state structure of the carbon; for example, magnesium has been used as reducer in combustion synthesis to produce exfoliated graphite with teflon^46^, few-layer graphene with CO^47^ and CO_2_ (dry ice)^48^; it also is used to react with CaCO_3_ to produce few-layer graphene with a conventional calcination method^49^. However, to the best of our knowledge, our group firstly developed the high-throughput SHS method for synthesizing few-layer graphene and doping nitrogen into few-layer graphene^50^,^51^.

Here, we prepared four few-layer graphene samples with different nitrogen contents using a patented method based on self-propagating high-temperature synthesis (SHS). We are particularly interested in the N-doped graphene samples because of its unexpected magnetic properties without the addition of 3*d* and 4*f* elements. N-doped graphene samples produced by SHS method exhibit both high Curie temperatures (higher than room temperature) and high coercive force. Thus, it is considered a good candidate for nanomagnetic applications.

## Experimental

### Synthesis of N-doped graphene

Magnesium (200 mesh, 99.0%), calcium carbonate (CaCO_3_, 99.5%), carbamide, (CO(NH_2_)_2_, 99.5%) and carbon dioxide (99.9% purity) were used as starting materials. These materials were purchased from Sinopharm Chemical Reagent Co., Ltd.

**Figure 1:**
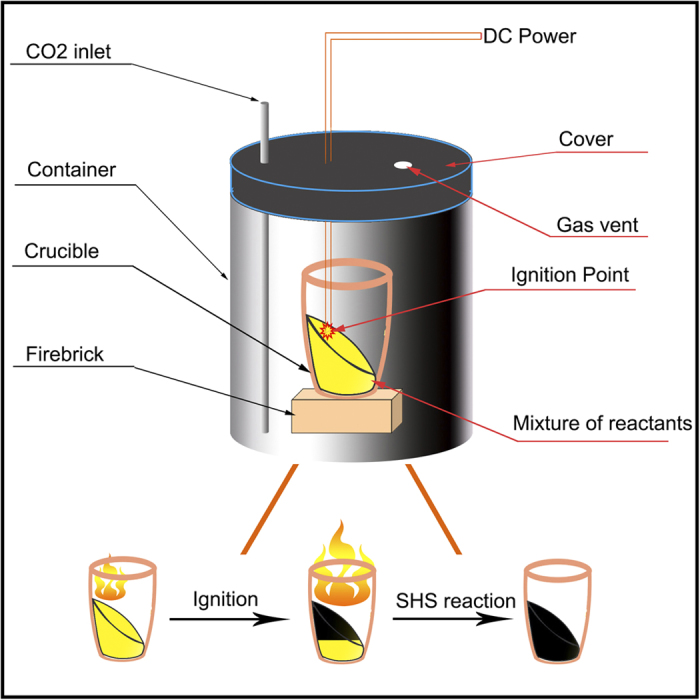
Schematic diagram of the reaction device.

Three N-doped graphene samples (Sample 1 to 3) with different nitrogen contents were synthesized by the SHS patent method^50^. Schematic diagram of the reaction device is shown in Fig. 1. In a typical preparation process for Sample 1, 8 grams of carbamide (30 grams for Sample 2 and 21 grams for Sample 3) were added to the mixture powder of 14.4 g of magnesium and 33.3 g of calcium carbonate and then milled in a mortar for 20 minutes. The reaction proceeded in a crucible which was put into a 21.2 L steel container under an atmosphere of carbon dioxide at atmospheric pressure, as shown in Fig. 1. The mixture of the reactants was ignited by an electric ignition device which is composed of a DC power source and a resistance-based wire heater using an ignition current of 22 A. The reaction spontaneously propagated through the mixture in the crucible from the upside to the bottom in a type of combustion wave and terminated when the combustion wave reached the bottom. A black raw product was then purified by dilute hydrochloric acid (10 v/v %) and stirred for two hours to remove the MgO, CaO and the remaining Mg metal. The product was then filtered, washed with deionized water and absolute ethanol. The thinner sheets in the product were further separated by using a centrifuge at 1000 rpm for 30 minutes and filtering the supernatant. Finally, the sample was dried in a vacuum at 120 °C for 24 hours. As a comparison, a pristine graphene sample (Sample 0) was also prepared by SHS from 16 grams of magnesium and 33.3 grams of calcium carbonate, which was based on the stoichiometric ratio of the reaction: 2 Mg + CaCO_3_ = 2 MgO + CaO + C; no carbamide was introduced in the reaction and the product was treated with the same processes as above mentioned.

### Characterization techniques

The phase composition of the as-prepared powders was analyzed by powder X-ray diffraction (XRD) analyses (Philips X’ Pert diffractometer) with CuKα radiation. Environmental scanning electron microscopy (ESEM, Helios Nanolab 600i) and high-resolution transmission electron microscopy (HRTEM JEM-2100) were used to observe the morphology of the graphene sheets. The TEM specimens were prepared by dropping ethanol/water (38 v/v%) solution containing 1 wt % N-doped graphene onto a copper grid and drying at 100 °C. Raman spectra were obtained using a Raman Station (B&WTEK, BWS435-532SY) with a 532 nm wavelength laser corresponding to 2.34 eV, 30% of the laser power (total power: 240 mW) was used on the samples. X-ray photoelectron spectroscopy (XPS, Thermo Fisher) was utilized to determine the bonding characteristics of the samples. All XPS peaks are calibrated according to the C 1 s peak (284.6 eV). The composition was confirmed using X-ray fluorescence (XRF, AXIOS-PW4400) in order to determine the presence of any metallic elements. 2 milligrams of N-doped graphene powder was spread out on the surface of boric acid powder (99.0%, Sinopharm Chemical Reagent Co., Ltd.), the effective test zone is a disk surface with a diameter of 20 mm. The magnetic properties were measured using a Quantum Design MPMS magnetometer based on a superconducting quantum interference device (SQUID).

## Results and Discussion

### Microstructure

**Figure 2:**
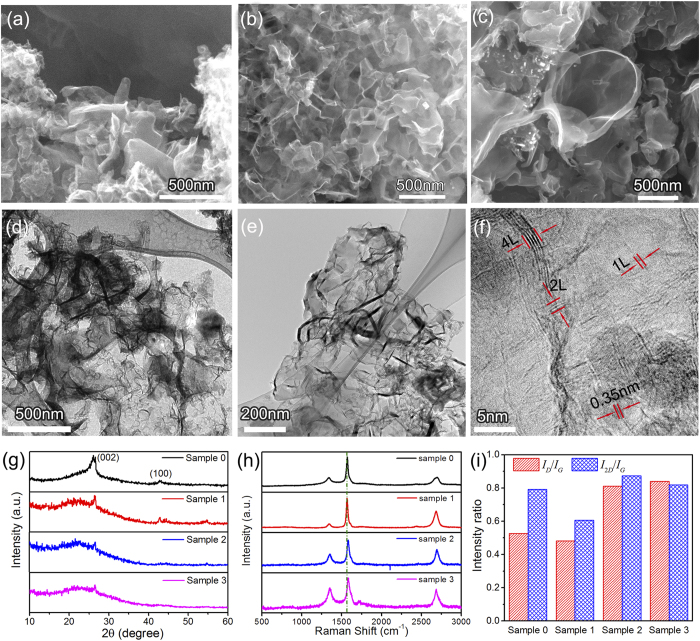
Characterizations of N-doped graphene samples. (**a**–**c**) SEM images of the pristine and N-doped graphene Sample 0, Sample 2 and Sample 3, respectively. (**d**) TEM image of Sample 0. (**e**) TEM image of Sample 2. (**f**) HRTEM of Sample 2. (**g**) XRD of the pristine and N-doped graphene samples. (**h**) Raman spectra of the pristine and N-doped graphene samples. (**i**) The intensity ratios of *I*_D_/*I*_G_ and *I*_2D_/*I*_G_ of the pristine and N-doped graphene in the Raman spectra.

Fig. 2(a-c) shows the morphology of pristine graphene and N-doped graphene sheets probed by SEM. The pristine graphene and N-doped graphene sheets have a wrinkled and 3D continuous structure. The 3D structure in Fig. 2(a) is composed of many tiny sheets; for N-doped graphene sheets with higher content of nitrogen (Sample 2 and Sample 3), the sheets are curved and more expansive, as shown in Fig. 2(b) and Fig. 2(c).

TEM observations were used to further investigate the high magnification morphology and crystalline structure of the pristine graphene and N-doped graphene sheets, and the typical TEM image of the pristine graphene sheet presents in Fig. 2(d). As observed in Fig. 2(d), the graphene sheets are thin and crinkly. In the TEM and HRTEM image of the N-doped graphene (Sample 2), abundant edges of the sheets can be seen. The layer number of N-doped graphene generally ranges from 1 to 5 according the HRTEM observation in Fig. 2(f).

The XRD patterns of N-doped graphene in Fig. 2(g) show a broad diffraction peak at about 25.9° which corresponds to the (002) diffraction of few layer graphene, and for Sample 0 and 1, the diffraction peak at 42.7° is the characteristic of the graphite of (100) diffraction. The weak peak intensity in Fig. 2(g) suggests that the average layer number of the graphene sheet obtained is less than that obtained before using similar method^48^,^52^.

The Raman spectrums of pristine and N-doped graphene sheets are shown in Fig. 2(h), and three peaks arising from graphene can be clearly found in the spectrums. The peaks at around 1585, 1340 and 2677 cm^−1^ are corresponding to the G-band, D-band and 2D-band, respectively, according to previous study^53^,^54^. In Raman spectroscopy, the D-band, known as the disorder or defect mode, originates from edge configurations in graphene where the planar sheet configuration is disrupted^55^,^56^. While the G band is the result of the first-order scattering of the E_2g_ mode of sp^2^ carbon domains. Both bands can be influenced by doping. D bands have been significantly enhanced in Sample 2 and Sample 3 with respect to pristine graphene and Sample 1, because pyrrolic and pyridinic N are usually accompanied by defects or edges in the graphene sheets. It is interesting to note that the G band of Sample 2 and Sample 3 moves to higher frequency which is sign of high density N doping; in the work of Zhao *et al*., a similar trend of the G band was also observed^57^.

Both 2D-and G-band features are highly relevant to layer numbers and defects. The D-band and G-band intensity ratio (*I*_D_/*I*_G_) is a measure of defect quantity^58^. As-prepared samples exhibit a broader peak at 2677 cm^−1^, which is a feature of graphene^19^,^59^,^60^,^61^. As shown in Fig. 2(i), the value of *I*_D_/*I*_G_ in graphene increases with the increasing of carbamide in the reactants, which indicates that the defect concentrations in N-doped graphene increase with the increasing of the N-content in the samples. This is due to the fact that the N atoms break the sp^2^ C six-member ring structure in graphene to form pyrrolic or pyridinc nitrogen^62^. The 2D-band and G-band intensity ratio (*I*_2D_/*I*_G_) can be used to estimate the layer number of graphene sheet, the values of *I*_2D_/*I*_G_ for both Sample 2 and Sample 3 are higher than that of Sample 1, indicating thinner thicknesses for Sample 2 and Sample 3. In addition, pyrrolic N bonding configuration also attenuates the 2D mode, as the intensity of 2D mode is dependent on the electron-doping concentration, which results in the inversion relationship of the *I*_2D_/*I*_G_ and the electron concentration. As a result, the reason for the value of *I*_2D_/*I*_G_ for Sample 3 lower than that of Sample 2 may be caused by the higher concentration of pyrrolic N in Sample 3 (also see the XPS analysis).

### Chemical features

**Figure 3:**
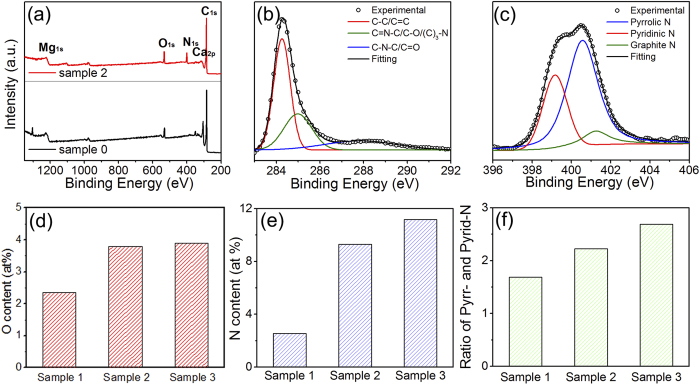
(**a**) XPS survey spectrum of Sample 0 (black) and Sample 2 (red). (**b**) XPS C 1 s spectrum and (**c**) XPS N 1 s spectrum of Sample 2. (**d**) O-contents in N-doped graphene samples. (**e**) N-contents in N-doped graphene samples. (**f**) Ratios of pyrrolic N and pyridinic N for N-doped graphene samples.

XPS is a common technique to measure the doping concentration and the N bonding configuration in N-doped graphene. As shown in Fig. 3(a), the XPS spectrum of pristine graphene and N-doped graphene shows a predominant C 1s peak at around 284.4 eV, a weak O 1 s peak at around 532.0 eV, Ca 2p peak at around 313.1 eV, Mg 1 s peak at around 1225.1 eV, and a pronounced N 1 s peak at around 400.0 eV in N-doped graphene. The spectrum was analyzed by X-peak software and corrected for the background signals using the Shirley algorithm prior to curve resolution^63^.

The C 1 s peak of the samples in XPS can be split into three peaks, which locate at 284.3, 285.0 and 287.9 eV (Gaussian decomposition and Lorentz decomposition are employed in this fitting). The main peak at 284.3 eV corresponds to graphite-like sp^2^-hybridized carbon (C-C)^53^,^64^. The secondary peak at 285.0 eV is assigned to sp^3^ hybridized C atoms bonded with O, N or (C)_3_-N bonds, which could be originated from C-O, pyrrolic or graphitic N bonding configuration. Furthermore, different from the N-doped graphene directly grown by CVD system, a new weak peak located at 287.9 eV can be observed in our sample. This peak indicates sp^2^ hybridized C atoms bonded with N, C-N-C bonds, which is originated from either graphitic or pyridinic N bonding configuration^65^. Similarly, the high resolution XPS spectrum in Fig. 3(c) of the N 1 s peak at 398.3 eV is assigned to sp^2^ hybridized aromatic N with two sp^2^ hybridized C neighbors in the form of C = N-C (pyridinic N) and the peak at 400.4 eV is assigned to the tertiary N in the form of N-(C)_3_ or H-N-(C)_2_ (pyrrolic N). These assignments are consistent with previous report^66^. Since the peak position of another possible N bonding configuration (graphitic N bonding, sp^2^ hybridized N atoms with three sp^2^ hybridized C neighbors) in the N-doped graphene lattice would appear around 402.0 eV, it indicates graphitic N bonding configuration is very limited in our N-doped graphene. The pyridinic bonding configuration refers to the bond between N and two C atoms at the defects or edges of the N-graphene. The pyrrolic N bonding refers to the N atom bonding in a five-membered ring structure. Compared with the XPS spectrum captured for the pristine graphene (Sample 0), these changes observed for the C 1 s orbit in the N-doped graphene suggest that the N doping behavior indeed occurs in graphene lattice to a certain extent.

On the basis of the XPS analyses, the N and O contents of N-doped graphene samples are shown at Fig. 3(d,e). The N contents are about 2.53 at.%, 9.21 at.% and 11.17 at.% and the O contents are about 2.32 at.%, 3.79 at.%, 4.02 at.% for samples 1, 2 and 3, respectively. The ratios of pyrrolic nitrogen versus pyridinic nitrogen are shown in Fig. 3(e). It can be seen that with the nitrogen content increases from 2.53 to 11.17 at.%, the ratio of pyrrolic versus pyridinic N also increases from 1.69 to 2.67.

### Magnetic properties

**Figure 4:**
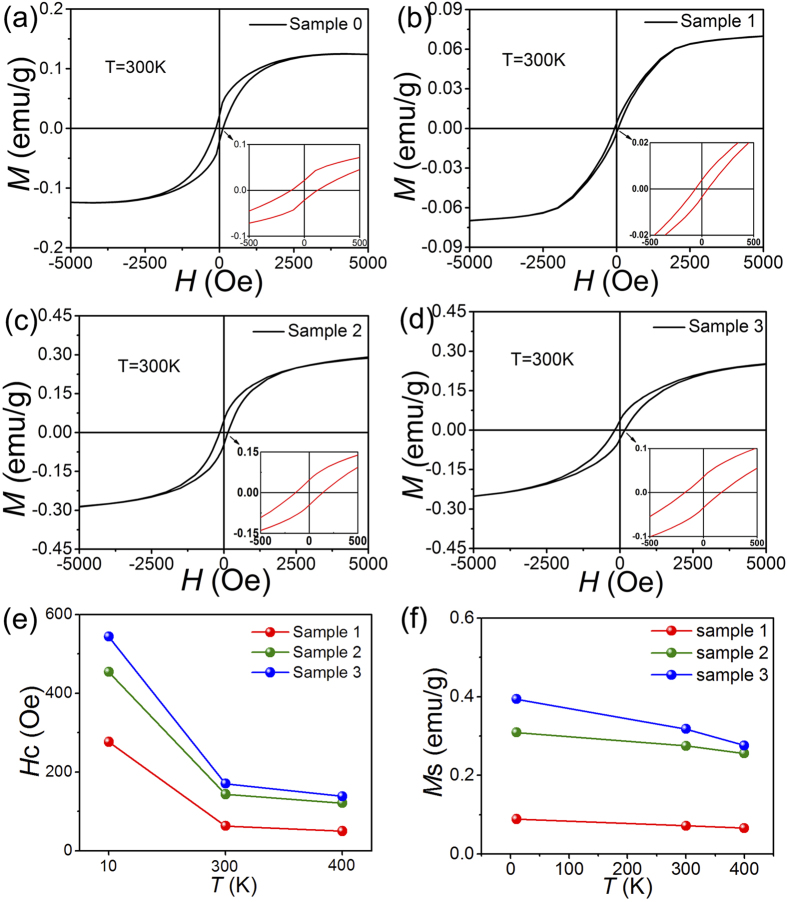
(**a**–**d**) M versus H curves of the pristine and N-doped graphene at room temperature. (**e**) *H*c of N-doped graphene at 10 K, 300 K and 400 K. (**f**) *M*_s_ of N-doped graphene at 10 K, 300 K and 400 K.

The magnetization versus magnetic field (*M*-*H*) curves for pristine and N-doped graphene samples measured at room temperature (300 K) are shown in Fig. 4(a–d). All magnetic data are corrected for the background signal from the sample holder.

The magnetic hysteresis loops shown in Fig. 4(a–d) clearly indicate the ferromagnetism of pristine and N-doped graphene sheet. The coercive forces (*H*_c_) and saturation magnetizations (*M*_s_) can be obtained from the magnetic hysteresis loops in Fig. 4(a–d). The values of *M*_s_ at 10 K for Sample 0, 1, 2 and 3 are 0.072, 0.275 and 0.318 emu/g, respectively; and the values of *H*_c_ at room temperature for Sample 1, 2 and 3 are 63.1, 143.7 and 168.8 Oe, respectively. It is strange to see that the *H*_c_ and *M*_s_ and of Sample 0 (0.125 emu/g and 117.8 Os) are higher than those of Sample 1 and lower than those of Sample 2 and 3, which means that the pristine graphene has higher ferromagnetism than the sample with low content of N (2.53 at%). The result can be understood considering the result in Fig. 2(i), which shows that the ratio of I_D_/I_G_ for the pristine graphene is also higher than that of Sample and lower than those of Sample 2 and 3. Since it is well accepted that defects play the main role in the ferromagnetism and the ratio of I_D_/I_G_ is positively corresponding to defect concentration, the higher ferromagnetism for Sample 0 can be understood from the point of view of defect concentration.

This result also suggest that since the introduction of carbamide can change the reaction temperature (large amount of energy is needed for the decomposition of carbamide, and the reaction enthalpy changes for the reaction with and without carbamide are quite different) and the reaction atmosphere, there may be differences in the mechanism for the two reactions with and without carbamide. As a result, Sample 0 only has limited reference value.

The coercive forces and the saturation magnetizations at different temperature are shown in Fig. 4(e,f). It can clearly be seen that the coercive forces of the three N-doped samples increase with the increasing of N content in N-doped graphene at each temperature. The coercive forces reach 544.2 Oe at 10 K and 168.8 Oe at 300 K for Sample 3 with the highest N content. The coercive forces decrease with the rise of temperature for the three samples.

The values of *M*_s_ of the four samples have similar trends with those of the coercive force. They decrease with the increasing of N content and temperature. The values of *M*_s_ reach 0.282 emu/g at 10 K and 0.148 emu/g at 300 K for Sample 3, which are comparable to graphene obtained in freestanding MoS_2_ nanosheet or other dopant-free diluted magnetic semiconductors^67^. The key finding in our case is the experimental observation of ferromagnetism in metal-free N-doped graphene at different temperatures. The results clearly show that the emergence of ferromagnetism and the values of the coercive force and remanent magnetization are positively correlated with the N content in N-doped graphene.

XRF measurement is utilized to check the content of the ferromagnetic impurities in our samples. The specific ferromagnetic impurities are 8.9 ppm Fe and 5.0 ppm Ni as shown in Fig. S3, so the total content of ferromagnetic impurities in N-graphene is 13.9 ppm, which is no more than 15 ppm. If it is assumed that all the ferromagnetic impurities present in the form of bulk Fe metal whose magnetization is 217.6 emu/g at room temperature^68^, the expected ferromagnetic contribution is calculated as 0.0033 emu/g for 15 ppm of Fe, which is neglectable. This indicates that *d* or *f* element is not responsible for the observation of ferromagnetism in N-doped graphene. Therefore the presence of defects seems to be the main factor in the emergence of ferromagnetism in N-doped graphene.

In addition, it was reported that Pyrrolic N-doped graphene can be produced based on a stoichiometric dehalogenation of perhalogenated arene and pyridine precursors by a transition metal^22^ and through hydrothermal^5^. Our work suggests that the SHS method may also be a good candidate to produce pyrrolic N doped graphene with high N content.

**Figure 5:**
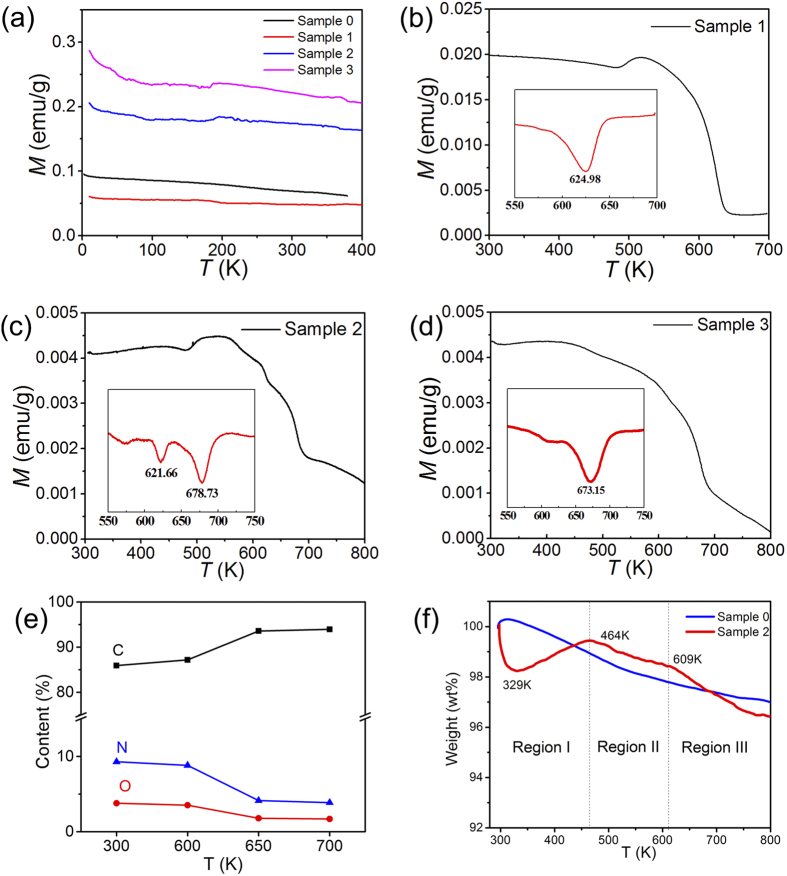
Magnetic susceptibility versus temperature for the pristine and N-doped graphene. (**a**) Measured between 10 K and 400 K, H = 3000 Oe. (**b**) Measured between 300 K and 700 K, H = 1000 Oe. (**c**,**d**) Measured between 300 K and 800 K, H = 500 Oe. The insets are the derivatives of *M*_s_ with respect to temperature. (**e**) The contents of C, O and N for Sample 2 heat-treated at different temperatures. (**f**) Thermogravimetric curves of Sample 0 and Sample 2.

The magnetization behaviors of the four samples recorded at 3000 Oe are shown in Fig. 5(a). It can be seen that all of the samples exhibit ferromagnetism and that the saturation magnetization don’t change obviously in the temperature range 10–400 K. In order to determine the Curie temperature of N-doped graphene, the magnetization behaviors were further measured in the temperature range 300–800 K at 1000 Oe for Sample 1 and at 500 Oe for Sample 2 and 3, and the corresponding *M* ~ *T* curves are shown in Fig. 5(b–d). The derivatives of *M* ~ *T* curve with respect to temperature range 550–700 K are plotted as the insets in Fig. 5(b–d) to determine more accurately the magnetization changes of the samples. The inset shows only one big and broad peak at 625 K for Sample 1. In contrast, Sample 2 shows a middle peak at 621 K and a big peak at 678 K, while Sample 3 shows a small peak at 620 K and a big peak at 673 K. These peaks reflect the drop of magnetization with the increase of temperature which may offer information on the Curie temperatures or the structure change of the materials.

To study the reason for the decreasing magnetism, take Sample 2 as an example, we have heated the Sample 2 in vacuum for 5 minutes at 600 K, 650 K and 700 K, respectively. XPS measurements were conducted for the samples heated at different temperature, the contents of C, O and N for Sample 2 were shown in Fig. 5 (e). We can know that the relative content of carbon increases while the relative contents of nitrogen and oxygen decrease with the increasing of temperature from 300 to 650 K; the contents of C, O and N become stable between 650 and 700 K. The XPS results of the samples after heat treatment indicate that a transformation process happened between 600 and 650 K which is mainly corresponding to the loss of N element and partly related with the loss of O element in Sample 2.

To further support the XPS results, thermogravimetric (TG) analysis was conducted for Sample 0 and Sample 2. The TG curves are shown in Fig. 5(f). For Sample 0, the weight change is gently, while the weight change of Sample 2 is relatively notable. It can be seen that the TG curve of Sample 2 can be divided into three regions. For Region I (300 to 464 K), the weight of the Sample 2 decreases sharply and increases gradually, demonstrating a special desorption and adsorption process. For Region II (464 to 609 K), the slope of Sample 2 is similar to that of Sample 0, which indicates that the two samples experience similar process. For Region III (609 to 700 K), a quick weight loss happens for Sample 2 while the weight loss of Sample 0 is not obvious. So the decomposition of N-doped graphene happened after 609 K. This result is well consistent with the XPS results in Fig. 5(e) which also suggest that the loss of N element happens between 600 and 650 K.

Connected these results with the behaviors of magnetism of the samples, it is reasonable to conclude that the peaks at 625 K for the N-doped graphene samples are caused by the decomposition of N groups, with the loss of N groups which are the source of magnetic moment, the magnetization drops quickly. So the peak at 625 K is the result of thermal instability for N-doped graphene between 600 and 650 K.

After 650 K, the relative contents of C, N and O are stable as shown in Fig. 5(e), the magnetization changes of the samples around 678 K are corresponding to Curie temperatures, which are 678 K for Sample 2 and 673 K for Sample 3. For Sample 1, it is clear that the magnetization change caused by N group decompose and Curie transformation are overlapped, so its Curie temperature is in the temperature range of 609–650 K.

As above mentioned, the N plays an important role in the magnetic properties of N-doped graphene, both the *H*_c_ and *M*_s_ increase with the increase of N content in N-doped graphene. However, it is interesting to find that the Curie temperature of Sample 3 is a little less than that of Sample 2. We would discuss the meaning of the phenomenon.

In the case of Curie temperature, although it is not clear why there is such strong interactions in these N-doped samples, which give rise to high Curie temperatures, we could speculate and discuss the influence factors of Curie temperature according to the well known physical principles, that is the magnetic response of the N-doped graphene could be determined by the competition between the RKKY interactions and the screening effect of electrons. We will discuss the issue from two aspects.

Firstly, we should point out that pyrrolic N plays an important role in the formation of magnetic moments, because pyrrolic N can induce a net magnetic moment of 0.95 μ_B_/N as proposed by Li *et al*.^21^ and pyrrolic N has been proved to be the main defects in our N-doped graphene; while pyridinic N and graphitic N have less influence on the spin polarization. In addition, the pyrrolic N bonding is usually accompanied by the generation of a large amount of other defects such as vacancy, disorder and edge defects in N-doped graphene lattice, so other kinds of defects can also act as the source of localized magnetic moments^13^,^14^. Furthermore, it should be mentioned that the carbon defects produced during SHS process also play a role in the formation of magnetic moments since Sample 0 exhibits ferromagnetism at room temperature.

Secondly, the ferromagnetism in N-doped graphene indicates that localized magnetic moments coming from pyrrolic N and defects can cause the ferromagnetism response by the magnetic coupling. This coupling effect could be realized through the RKKY interactions by delocalized electrons^32^,^35^. In general, with the increasing of defect concentration, the distance between the magnetic moments from the defects decreases, as a result the RKKY interactions can be enhanced. However, when the defect concentration is high, screening effect from the electrons must be considered, which can weaken the interaction^69^. As a result, with the N content increasing, the interaction between magnetic moments becomes strong, and the Curie temperature rises; however, the screen effect weakens the interaction when the defect concentration is too high, the Curie temperature decreases. So the Curie temperature should have a threshold value, corresponding to a threshold of N concentration. With respect to our N-doped graphene, the Curie temperature of Sample 3 is a little lower than that of Sample 2 indicates that the threshold of N concentration may be below 11.17 at.%.

It is worth emphasizing that the SHS method plays an important role in the formation of pyrrolic N and defects in the N-doped graphene. SHS method used in the present study is a process far from equilibrium technique, which utilizes the energy released by the exothermic combustion reactions of the starting materials; the combustion reactions can produce very high temperature instantly (up to 4000 K and generally higher than 2000 K) and then cool quickly^12^; the reaction time for the samples in this work is about 40 seconds. Our work suggests that SHS exhibits a good prospect for the high-throughput production of carbon-based ferromagnetic materials with high and controllable magnetic properties.

## Conclusion

In summary, we have produced few layer pristine and N-doped graphene by SHS method. Room temperature ferromagnetism has been found and related to carbon defects and pyrrolic N arising from nitrogen doping in N-doped graphene. It has also been found that the saturation magnetization and coercive field increase with the increasing of nitrogen content in the samples. The Curie temperature is 673 K~678 K for the N-doped graphenes with higher N contents. The drop of magnetism between 600 and 650 K is caused by the thermal instability of N-doped graphene. N-doped graphene samples produced by SHS method exhibit both high Curie temperatures and high coercive force. The work proves that SHS method is a promising high-throughput method to produce N-doped graphene which may have potential applications in electromagnetism.

## Additional Information

**How to cite this article**: Miao, Q. *et al*. Magnetic properties of N-doped graphene with high Curie temperature. *Sci. Rep*. **6**, 21832; doi: 10.1038/srep21832 (2016).

## Supplementary Material

Supplementary Information
