# Supplementary material for: Magnetic properties of N-doped graphene with high Curie temperature
Source: Sci Rep. 2016 Feb 24;6:21832. doi: 10.1038/srep21832 (PMC4764835; doi:10.1038/srep21832)
Supplement: Supplementary Information [file srep21832-s1.pdf]

Supplementary Information

Magnetic properties of N-doped graphene with high Curie temperature

*Qinghua Miao,Lidong Wang, Zhaoyuan Liu, Bing Wei , Fubiao Xu, and Weidong Fei**

School of Materials Science and Engineering, Harbin Institute of Technology, Harbin150001, China

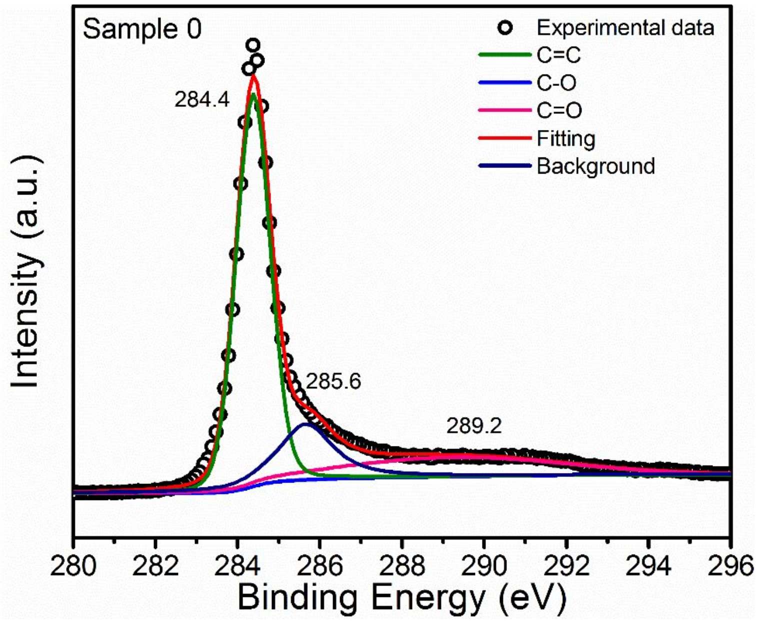

Figure S1 XPS C 1 s peak of pristine graphene

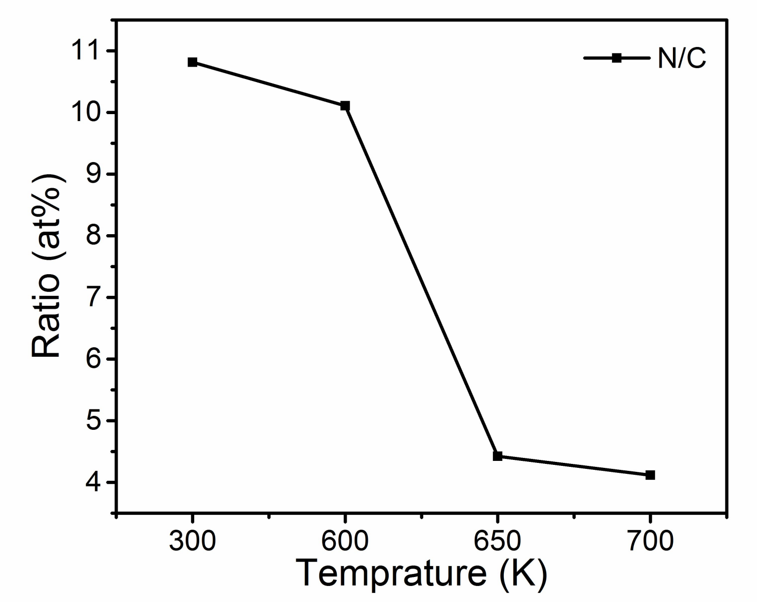

Figure S2 Ratio of N/C after annealing at different temperature of Sample 2

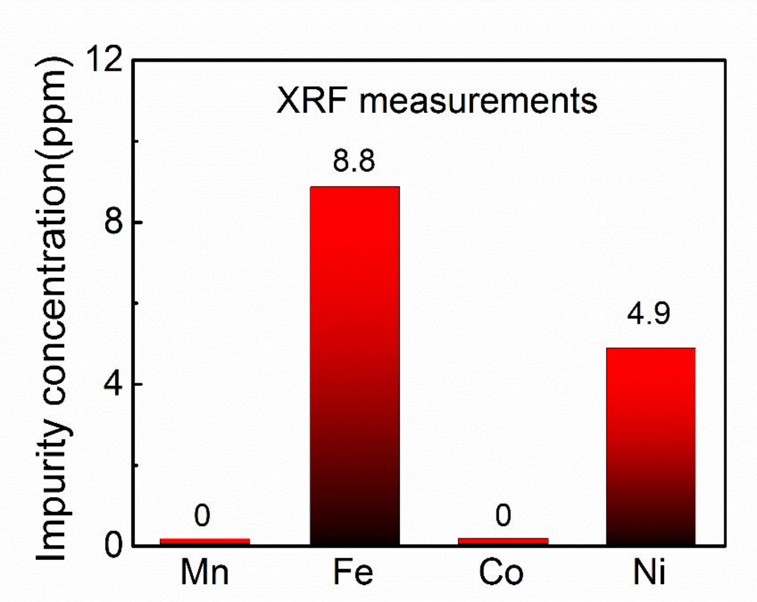

Figure S3 Content of ferromagnetic impurities of sample 2 by XRF

Table S1 XPS of Sample 2 treated by different temperature

T(K) / PP At%	300	600	650	700
C	85.91	87.17	93.57	93.94
N	9.29	8.81	4.14	3.87
O	3.79	4.02	2.29	2.2
